# Intermittent low-dose digoxin may be effective and safe in patients with chronic heart failure undergoing maintenance hemodialysis

**DOI:** 10.3892/etm.2014.2013

**Published:** 2014-10-09

**Authors:** XIAOZHAO LI, XIANG AO, QIONG LIU, JINGHUA YANG, WEISHENG PENG, RONG TANG, YONG ZHONG, TING MENG, LU GAN, QIAOLING ZHOU

**Affiliations:** 1 Department of Nephrology, Xiangya Hospital of Central South University, Changsha, Hunan 410008, P.R. China; 2 Department of Cardiology, Xiangya Hospital of Central South University, Changsha, Hunan 410008, P.R. China

**Keywords:** chronic heart failure, digoxin, heart function, maintenance hemodialysis

## Abstract

A low dose of digoxin is known to reduce mortality and hospitalization in patients with heart failure; however, the safety of digoxin in treating patients with heart failure on maintenance hemodialysis remains controversial. The objective of this study was to determine the effectiveness and safety of digoxin at lower doses in patients with heart failure on maintenance hemodialysis using a retrospective cohort study. This study included 67 heart-failure patients on maintenance hemodialysis: Twenty-four patients received intermittent low doses of digoxin (ILDD), 23 patients received continuous low doses of digoxin (CLDD) and the remaining patients were used as a control group without digoxin treatment. The brain natriuretic peptide (BNP) level and serum digoxin concentrations (SDCs) were measured by ELISA and the changes in left ventricular end diastolic diameter (LVEDD), left ventricular ejection fraction (LVEF), cardiac output (CO) and heart rate (HR) were evaluated by two-dimensional echocardiography. The symptoms of digoxin toxicity were monitored in the treated patients. Compared with the control group, LVEDD, BNP and HR decreased significantly between days 0 and 60 in the ILDD and CLDD groups, but LVEF and CO increased between days 0 and 60 in the same groups (all P<0.05). The levels of BNP and the LVEDD, CO, LVEF and HR were not significantly different between the ILDD and CLDD groups (P>0.05). Furthermore, and the mean SDC of the ILDD group was lower than that of the CLDD group. In the ILDD group, no patients had apparent symptoms of toxicity, but four patients developed digoxin toxicity in the CLDD group. In conclusion an intermittent lower dose of digoxin has beneficial effects and clinical safety in hemodialysis patients with congestive heart failure.

## Introduction

Chronic heart failure (CHF) is one of the most frequent complications in patients with chronic kidney failure receiving long-term hemodialysis. Despite the significant mortality associated with heart failure, there are limited therapeutic options proven to prevent and treat heart failure in patients on dialysis. Digoxin is one of the most commonly prescribed drugs for the treatment of CHF but, as a substantial fraction of the absorbed dose is cleared by the kidneys, its toxicity is often the result of an impaired renal function (1). Digoxin has a small therapeutic-to-toxic margin in patients with CHF (2), particularly in those patients who often have renal dysfunction, and it is therefore logical that, in recent years, numerous physicians have stopped using digoxin for the treatment of patients with CHF on hemodialysis.

Accumulative evidence indicates that digoxin significantly reduces the primary combined end-point of all-cause mortality or cardiovascular hospitalization in elderly patients with heart failure at low doses and low serum digoxin concentrations (SDCs) (3–5). The National Kidney Foundation Kidney Disease Outcomes Quality Initiative also included digoxin in its end-stage renal disease cardiovascular disease guidelines for the treatment of cardiomyopathy and atrial fibrillation (6). On the basis of such evidence, several studies have attempted to verify the safety of digoxin and how the drug should be properly managed and monitored in patients with chronic renal insufficiency (4,7). Ahmed *et al* (4) reported that a low dose (125 μg every other day) may be preferable in frail, elderly heart-failure patients with impaired kidney function; however, Chan *et al* (7) reported that digoxin use at a similar dose among patients who were on hemodialysis was associated with an increased mortality, particularly among those with low predialysis K^+^ concentrations. There is, therefore, uncertainty about the long-term efficacy and safety of digoxin in patients with heart failure undergoing maintenance hemodialysis. Owing to the discrepancy in the results of the above studies, the objective of the present study was to determine the effect of digoxin at lower doses (62.5 μg every other day) on heart function and safety in heart-failure patients on maintenance hemodialysis.

## Materials and methods

### Study design

A retrospective cohort study was conducted to evaluate the effectiveness and safety of lower doses of digoxin in dialysis patients with symptomatic heart failure and normal sinus rhythm. Patients received two different doses of digoxin (62.5 μg every day and 62.5 μg every other day) or received no digoxin as a matching disease control group. The study was in compliance with the Declaration of Helsinki and was approved by the Ethics Committee of Xiangya Hospital of Central South University (Changsha, China). Informed consent was obtained from each patient.

### Study patients

A total of 67 patients with CHF on maintenance hemodialysis who were from the Renal Division, Xiangya Hospital of Central South University and who underwent a 4-h hemodialysis session five times every two weeks between September 2010 and September 2013 were included in this study. The patients fulfilled the 1928 New York Heart Association Functional Classification (NYHA) revised criteria for CHF (8). Inclusion criteria for this study were symptomatic heart failure with NYHA functional classification class II–IV, left ventricular ejection fraction (LVEF) of ≤45%, normal sinus rhythm and aged ≥50 years. Exclusion criteria were mainly associated with different types of cardiac arrhythmias. The patients returned for follow-up visits after 15, 30 and 60 days. Clinical and laboratory data of the patients were collected prior to digoxin therapy and at each follow-up visit. Clinical and laboratory examination, including SDCs, brain natriuretic peptide (BNP) levels, heart rates (HRs), blood pressure and echocardiography were performed at baseline and at each follow-up visit.

### Drug therapy

Twenty-four patients received 125 μg digoxin per day orally for three days and then 62.5 μg every other day [intermittent low doses of digoxin (ILDD) group]. Twenty-three patients received 125 μg digoxin per day orally for three days and then 62.5 μg per day thereafter [continuous low doses of digoxin (CLDD) group]. Twenty patients who were not using digoxin were observed as a disease control (control group).

Several patients were taking angiotensin-converting enzyme inhibitors or angiotensin-receptor antagonists, or calcium-channel blockers and α- or β-blockers. Recombinant human erythropoietin (EPO) and calcitriol were administered to those patients. The EPO (3000U iH three times a week) was administered to dialysis patients to improve anemia, and Calcitriol (0.25 μg po. every day) increases blood calcium levels (Ca^2+^) of dialysis patients with low calcium and acts in concert with parathyroid hormone.

### Observation of the symptoms of digoxin toxicity

The symptoms of digoxin toxicity in the ILDD and CLDD groups were recorded and analyzed in every follow-up visit. These symptoms included loss of appetite, nausea, vomiting, diarrhea, headaches, blurred or yellowish-green vision, confusion, irregular heartbeat and fatigue.

### Two-dimensional echocardiography assessment and measurement of SDCs and BNP levels

Left ventricular end diastolic diameter (LVEDD), LVEF, cardiac output (CO) and HR were assessed by two-dimensional echocardiography. ELISA was used to determine SDCs were quantified by the ELISA kit and this was performed according to the protocol provided by the supplier (Yueyan Biological Technology, Shanghai, China). The levels of plasma BNP were quantified by ELISA kits and performed according to the manufacturer’s instructions (Kaibo Biological Technology, Shanghai, China) in accordance with the manufacturer’s instructions. Following baseline assessment and the introduction of digoxin treatment, patients were monitored for 60 days, with follow-up visits and measurement of heart function, SDCs and BNP levels after 15, 30 and 60 days.

### Statistical analysis

Enumeration data were analyzed with the χ^2^ test, and measurement data were analyzed by one-way analysis of variance or repeated-measures analysis of variance, followed by Fisher’s protected least significant difference test. P<0.05 was considered to indicate a statistically significant difference. Analyses were conducted using SPSS version 17.0 (SPSS, Inc., Chicago, IL, USA).

## Results

### Characteristics of the study population

The demographic and clinical features of the patients with heart failure on maintenance hemodialysis are shown in Table I. In the present study, no significant differences were observed in the baseline characteristics among the different groups. The patients returned for follow-up visits on days 15, 30 and 60. In the period prior to the third follow-up visit, four patients terminated digoxin therapy and in four patients digoxin was stopped by the general practitioner due to presumed digoxin-related side effects (one patient had an irregular heart beat and three patients had minor gastrointestinal side effects).

### Effect of intermittent and continuous low doses of digoxin on the heart function of patients with heart failure on hemodialysis

**Figure 1:**
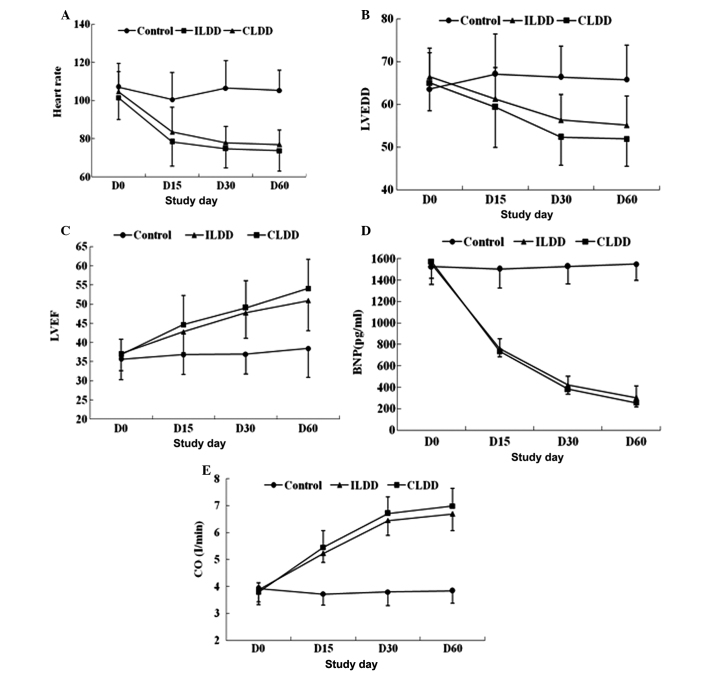
Effect of intermittent and continuous low doses of digoxin on the heart function of patients with heart failure undergoing maintenance hemodialysis. (A) Heart rate: ILDD/CLDD vs*.* control, P<0.001, ILDD vs. CLDD, P=0.072. (B) LVEDD: ILDD/CLDD vs. control, P<0.001, ILDD vs*.* CLDD, P=0.051. (C) LVEF, ILDD/CLDD vs. control P<0.001: ILDD vs. CLDD, P=0.149. (D) BNP: ILDD/CLDD vs. control, P<0.001, ILDD vs. CLDD, P=0.592. (E) CO: ILDD/CLDD vs. control, P<0.001: ILDD vs. CLDD, P=0.094. Data were analyzed by repeated-measures analysis of variance. ILDD, intermittent low doses of digoxin; CLDD, continuous low doses of digoxin; LVEDD, left ventricular end diastolic diameter; LVEF, left ventricular ejection fraction; BNP, brain natriuretic peptide; CO, cardiac output.

To investigate the effect of intermittent and continuous low doses of digoxin on the heart function of patients with heart failure on hemodialysis, the expression level of BNP was analyzed by ELISA and the changes in LVEDD, CO, LVEF and HR were assessed by two-dimensional echocardiography. According to repeated-measures analysis of variance, LVEDD, BNP levels and HR were significantly decreased between days 0 and 60 in the ILDD and CLDD groups compared with the values in the disease control group (all P<0.05). In contrast to the significant decrease in LVEDD, BNP levels and HR, the values of LVEF and CO were increased between days 0 and 60 in the ILDD and CLDD groups compared with the values in the disease control group (all P<0.05); however, over the entire time-period, the values of BNP, LVEDD, CO, LVEF and HR were not significantly different between the ILDD and CLDD groups (P>0.05) (Fig. 1).

### Symptoms of digoxin toxicity and SDCs following intermittent and continuous low-dose digoxin administration

To evaluate the safety of digoxin in patients with heart failure on maintenance hemodialysis, the most common symptoms of digoxin toxicity were monitored in the ILDD and CLDD groups. In the ILDD group, the 24 patients had no apparent signs or symptoms of toxicity; however, four patients developed definite digoxin toxicity in the CLDD group and digoxin administration was therefore discontinued in those four patients (Table II). The mean SDC was 0.55 ng/ml in the ILDD group, while in the CLDD group it was 0.71 ng/ml (Table III). Taken with previous results that increasing SDCs were associated with increased mortality (7), the findings suggested that intermittent low-dose digoxin is safer than continuous low-dose digoxin.

## Discussion

Digoxin, as an inexpensive drug, has been used for >200 years for the treatment of cardiovascular disease, and it still has an important place in the management of patients with CHF. A clinical study demonstrated reductions in one-year mortality when digoxin was used at a low SDC of 0.5–0.9 ng/ml, corresponding to ≤125 μg/day (9). Other retrospective analyses of the Digitalis Investigation Group trial also showed that digoxin used at similarly low SDCs had beneficial effects on morbidity and mortality (10,11); thus, low-dose digoxin has been clearly shown to have beneficial clinical effects.

Digoxin is eliminated from the body by the kidneys (12). The drug can remain in the body for 36–48 h in individuals with normal kidney function but may take between three and five days to clear in patients with renal insufficiency. This means that the drug may accumulate in patients with renal function impairment, with patients on dialysis being more likely to develop high SDCs and digoxin toxicity than other patients (12). Digoxin is mostly stored in the skeletal tissues rather than the blood and is not effectively removed by dialysis or exchange transfusion, which results in the underuse of digoxin in patients with heart failure on dialysis; therefore, evaluating the safety of prescribing digoxin is important for patients who are undergoing long-term renal replacement therapy.

Ahmed *et al* (4) reported that a low dose of 125 μg digoxin every other day may be the preferred option in frail, elderly heart-failure patients with impaired kidney function. Chan *et al* (7), however, studied 4,549 incident hemodialysis patients who were digoxin users, and the results showed that the median prescribed dosage was 62.5 μg/day (125 μg every other day) and the median serum level was 1.0 ng/ml within this particular cohort of digoxin users. In addition, digoxin use was associated with a 28% increased risk of mortality, while an increasing serum digoxin level was also associated with mortality. This increased mortality risk with serum digoxin level was most apparent in patients with lower predialysis serum K^+^ levels. These results suggest that the use of digoxin by patients who are on hemodialysis is associated with increased mortality, particularly among those with low predialysis K^+^ concentrations.

In the present study, the effect of ILDD on the heart function of dialysis patients with CHF was observed. The results showed that the LVEDD, BNP level and HR were significantly decreased while the LVEF and CO were increased between days 0 and 60 in the digoxin groups compared with those in the control group. No significant difference was found for the expression of BNP, LVEDD, CO, LVEF and HR between days 0 and 60 in the CLDD and ILDD groups. ELISA was subsequently used to measure SDCs, and showed that the median serum level was 0.55 ng/ml in the ILDD group and 0.71 ng/ml in the CLDD group. No evident digoxin intoxication was found in the ILDD group. These findings suggested that intermittent lower doses of digoxin are effective and safe in patients with CHF on hemodialysis.

According to the possible mechanistic explanations for digoxin action, digoxin not only directly inhibits the Na^+^/K^+^-ATPase pump in the membrane of the cardiac myocyte but also inhibits the rennin-angiotensin-aldosterone system by inhibiting the Na^+^/K^+^-ATPase in the renal tubules (4). The effects of digoxin have long been known to be dependent on digoxin dose and SDC (4); however, the favorable effects of digoxin at low SDCs on natural end-points, such as all-cause hospitalizations and mortality, are likely to be mediated via the neurohormonal-modulating properties of digoxin (4). It has been suggested that the neurohormonal properties of digoxin are best exerted at low SDCs (4).

In the present study, consistent with previous studies (4,7), it was demonstrated that a lower dose of digoxin could improve the heart function of patients on dialysis. Furthermore, the results indicated that ILDD and CLDD were similarly effective in patients on dialysis, with the SDC in the ILDD group being 0.55 ng/ml. Digoxin is a drug with a narrow therapeutic-to-toxic margin, whereby patient-level pharmacokinetics, metabolism and clearance factors aggregate to introduce variability in drug responsiveness (7). Higher SDCs have been shown to increase mortality, yet lower SDCs reduce morbidity and mortality (13). Chan *et al* (7) reported that each 1 ng/ml increase in SDC significantly increased the risk of mortality by 19%. The results in the present study showed that the SDC in the ILDD group was considerably lower than that in the CLDD group. The most common symptoms of digoxin toxicity were monitored in the ILDD and CLDD groups. In the ILDD group, the 24 patients had no apparent signs or symptoms of toxicity; however, four patients developed definite digoxin toxicity in the CLDD group. When these findings are considered together, an intermittent low dose of digoxin is believed to be safe and has clearly been shown to have beneficial clinical effects on patients with CHF undergoing hemodialysis.

In conclusion, despite the small sample in this study, the results of the present analysis demonstrate that the use of digoxin in intermittent lower doses plays an important role in improving quality of life and easing the burden on dialysis patients with heart failure. These data may provide a new stimulus to further evaluate the safety and effectiveness of lower-dose digoxin in dialysis patients with symptomatic heart failure through a large, multicenter randomized clinical trial in the future.

## Figures and Tables

**Table I:** Demographic and clinical features of the patient cohort.

Characteristic	Control, n=20	ILDD, n=24	CLDD, n=23
Male, n (%)	15 (75)	18 (75)	17 (74)
Age in years, mean ± SD	65±8	66±6	66±7
BMI in kg/m^2^, mean ± SD	22.0±3.1	21.2±2.8	21.5±2.6
Systolic BP in mmHg, mean ± SD	155±18	152±16	153±15
Diastolic BP in mmHg, mean ± SD	74±16	75±18	76±17
Cardiothoracic ratio >0.55, n (%)	20 (100)	24 (100)	23 (100)
NYHA class, n (%)			
II	9 (45)	11 (46)	11 (48)
III	10 (50)	12 (50)	11 (48)
IV	1 (5)	1 (4.2)	1 (4.3)
ACE-inhibitors user, n (%)	5 (25)	6 (25)	5 (22)
ARB antagonists user, n (%)	5 (25)	6 (25)	7 (30)
α-blockers user, n (%)	5 (25)	7 (29)	6 (26)
β-blockers user, n (%)	10 (50)	12 (50)	11 (48)
Statins user, n (%)	1 (5)	2 (8)	2 (8.7)
CCB user, n (%)	20 (100)	24 (100)	23 (100)
Nitrate user, n (%)	10 (50)	12 (50)	11 (48)
EPO user, n (%)	20 (100)	24 (100)	23 (100)
Calcitriol user, n (%)	20 (100)	24 (100)	23 (100)
History of glomerulonephritis, n (%)	14 (70)	17 (71)	16 (70)
History of hypertension, n (%)	4 (20)	5 (21)	5 (22)
History of diabetes, n (%)	2 (10)	2 (8.3)	2 (8.7)
Time on dialysis, weeks	5 (2)	5 (2)	5 (2)
Years on dialysis	5.8	5.9	6.0
Hemoglobin in g/dl, mean ± SD	90.5±21.1	89.8±20.6	90.1±20.3
Calcium in mmol/l, mean ± SD	2.09±0.27	2.06±0.25	2.04±0.23
Phosphorus in mmol/l, mean ± SD	2.06±0.71	2.03±0.69	2.08±0.70

ILDD, intermittent low doses of digoxin (patients were instructed to take 125 μg digoxin per day orally for three days and then 62.5 μg every other day); CLDD, continuous low doses of digoxin (patients were instructed to take 125 μg digoxin per day orally for three days and then 62.5 μg every day); Control, patients who were not using digoxin were used as a disease control; The dialysis patients of the control group also underwent a 4-h hemodialysis session five times every two weeks. BMI, body mass index; SD, standard deviation; NYHA, 1928 New York Heart Association; ACE, angiotensin-converting enzyme; ARB, angiotensin-receptor blocker; CCB, calcium-channel blocker; EPO, human erythropoietin.

**Table II:** Symptoms of digoxin toxicity in the ILDD and CLDD groups.

Symptoms	ILDD, n=24	CLDD, n=23
Loss of appetite, n (%)	0/24 (0)	1/23 (4.3)
Nausea, n (%)	0/24 (0)	1/23 (4.3)
Vomiting, n (%)	0/24 (0)	0/23 (0)
Diarrhea, n (%)	0/24 (0)	1/23 (4.3)
Dizziness and headaches, n (%)	0/24 (0)	0/23 (0)
Blurred or yellowish-green vision, n (%)	0/24 (0)	0/23 (0)
Confusion, n (%)	0/24 (0)	0/23 (0)
Fast or slow heart rate, n (%)	0/24 (0)	0/23 (0)
Irregular heart beat, n (%)	0/24 (0)	1/23 (4.3)
Feelings of a racing, pounding or forcefully beating heart, n (%)	0/24 (0)	0/23 (0)
Fatigue and weakness, n (%)	0/24 (0)	0/23 (0)

ILDD, intermittent low doses of digoxin (patients were instructed to take 125 μg digoxin per day orally for three days and then 62.5 μg every other day); CLDD, continuous low doses of digoxin (patients were instructed to take 125 μg digoxin per day orally for three days and then 62.5 μg every day).

**Table III:** Serum digoxin concentrations of the ILDD and CLDD groups.

Detection time	ILDD (ng/ml)	CLDD (ng/ml)
Digoxin use 15 days	0.55±0.18	0.73±0.17
Digoxin use 30 days	0.52±0.13	0.71±0.15
Digoxin use 60 days	0.57±0.11	0.69±0.18

ILDD, intermittent low doses of digoxin (patients were instructed to take 125 μg digoxin per day orally for three days and then 62.5 μg every other day); CLDD, continuous low doses of digoxin (patients were instructed to take 125 μg digoxin per day orally for three days and then 62.5 μg every day).
